# Constructing efficient bacterial cell factories to enable one‐carbon utilization based on quantitative biology: A review

**DOI:** 10.1002/qub2.31

**Published:** 2024-02-08

**Authors:** Yazhen Song, Chenxi Feng, Difei Zhou, Zengxin Ma, Lian He, Cong Zhang, Guihong Yu, Yan Zhao, Song Yang, Xinhui Xing

**Affiliations:** ^1^ School of Life Sciences Shandong Province Key Laboratory of Applied Mycology, and Qingdao International Center on Microbes Utilizing Biogas Qingdao Agricultural University Qingdao China; ^2^ Department of Chemical Engineering University of Washington Seattle Washington USA; ^3^ Key Laboratory of Systems Bioengineering Ministry of Education Tianjin University Tianjin China; ^4^ Key Laboratory of Industrial Biocatalysis Ministry of Education Department of Chemical Engineering Tsinghua University Beijing China; ^5^ Center for Synthetic and Systems Biology Tsinghua University Beijing China; ^6^ Institute of Biopharmaceutical and Health Engineering Tsinghua Shenzhen International Graduate School, and Institute of Biomedical Health Technology and Engineering Shenzhen Bay Laboratory Shenzhen China

**Keywords:** ^13^C‐metabolic flux analysis, methylotrophic cell factories, one‐carbon feedstock, quantitative biology

## Abstract

Developing methylotrophic cell factories that can efficiently catalyze organic one‐carbon (C1) feedstocks derived from electrocatalytic reduction of carbon dioxide into bio‐based chemicals and biofuels is of strategic significance for building a carbon‐neutral, sustainable economic and industrial system. With the rapid advancement of RNA sequencing technology and mass spectrometer analysis, researchers have used these quantitative microbiology methods extensively, especially isotope‐based metabolic flux analysis, to study the metabolic processes initiating from C1 feedstocks in natural C1‐utilizing bacteria and synthetic C1 bacteria. This paper reviews the use of advanced quantitative analysis in recent years to understand the metabolic network and basic principles in the metabolism of natural C1‐utilizing bacteria grown on methane, methanol, or formate. The acquired knowledge serves as a guide to rewire the central methylotrophic metabolism of natural C1‐utilizing bacteria to improve the carbon conversion efficiency, and to engineer non‐C1‐utilizing bacteria into synthetic strains that can use C1 feedstocks as the sole carbon and energy source. These progresses ultimately enhance the design and construction of highly efficient C1‐based cell factories to synthesize diverse high value‐added products. The integration of quantitative biology and synthetic biology will advance the iterative cycle of understand–design–build–testing–learning to enhance C1‐based biomanufacturing in the future.

## 1 INTRODUCTION

**FIGURE 1:**
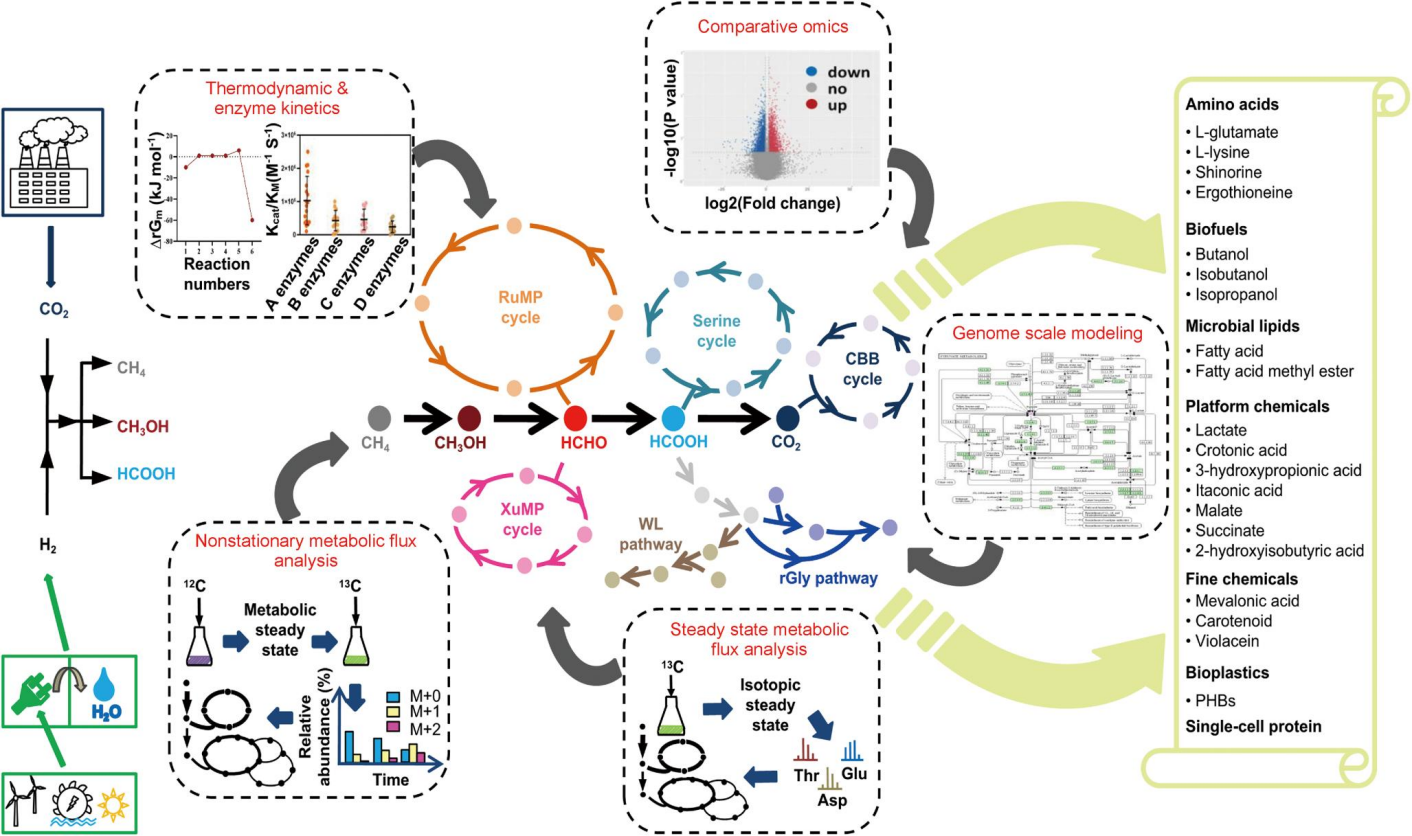
Quantitative technologies to guide the understanding, designing, and rewiring methylotrophic cell factories for high value‐added products synthesis from renewable one‐carbon feedstock. CBB, Calvin‐Benson‐Bassham; rGly, reductive glycine; RuMP, ribulose monophosphate; WL, Wood‐Ljungdahl; XuMP, xylulose monophosphate.

Utilizing one‐carbon (C1) feedstocks to produce bio‐based chemicals and biofuels plays an increasingly important role in solving resource and environmental problems, and thus shows strategic significance in building a carbon‐neutral, sustainable economic and industrial system. C1 feedstocks include various gaseous (e.g., methane, carbon dioxide, and carbon monoxide) and liquid (e.g., methanol, formaldehyde, and formic acid) compounds, and C1‐utilizing cell factories are microorganisms capable of utilizing these C1 feedstocks as the sources of carbon and energy [1]. To expedite the scaling up of C1‐utilizing processes, a two‐step strategy that couples carbon dioxide chemical catalysis with cell factory bioconversion is recently adopted [1, 2, 3, 4, 5, 6]. That is, hydrogen produced from water splitting with renewable energy (e.g., solar energy, wind energy, and tidal energy) [7] is used for the chemical reduction of carbon dioxide to generate organic C1 compounds (e.g., methane, methanol, and formate) [8, 9, 10] at large industrial scale (Figure 1). The resulting organic C1 compounds can then be efficiently converted into diverse products by engineered C1‐utilizing cell factories under mild reaction conditions [11, 12, 13].

Existing research of engineering C1‐utilizing cell factories involves three key aspects: (1) understanding the network and the basic principles of the metabolism of natural C1‐utilizing bacteria (e.g., methanotrophs, methylotrophs, and acetogens), (2) remodeling natural C1‐utilizing bacteria to improve their performance, and (3) engineering non‐C1‐utilizing well‐known bacteria or chassis cells (e.g., *Escherichia coli*) into synthetic strains that can use C1 feedstocks as the sole carbon and energy source. In this iterative cycle of understand–design–build–testing–learning, the quantitative prediction and assessment of metabolic processes on the genome scale is essential for developing new C1‐utilizing cell factories with improved industrial phenotypes [14, 15, 16, 17].

With the advancement of RNA sequencing technology and mass spectrometer (MS)‐based analysis, pioneering researchers have used quantitative multi‐omics extensively to study the metabolic processes of the C1 feedstock in natural C1‐utilizing bacteria [18, 19, 20, 21]; however, these tools cannot allow us to find the precursors to desired products or quantify flux distributions. Metabolic flux analysis (MFA) methods, including genome‐scale flux balance analysis (FBA) [22, 23], transient isotopic tracing analysis [24, 25], isotopically steady state MFA [26], and isotopically nonstationary MFA [27], have been applied in recent years to quantify distributions of carbon, energy, and reducing power during the oxidation and assimilation of C1 feedstocks into biomass in natural C1‐utilizing bacteria and non‐C1 bacteria (Figure 1). Findings from these quantitative analyses provide guidelines for systematically engineering both natural C1‐utilizing and non‐C1 microbial cells with established genetic tools and creating cell factories with desired phenotypes of growth and carbon conversion efficiency, as well as product titer, productivity, and yield [28]. The present review systematically summarizes the recent advances in the quantitative understanding and remodeling of the assimilative metabolic network of natural C1‐utilizing bacteria, as well as using quantitative data‐driven approaches to creating new C1‐utilizing bacterial chassis. The analyses will provide new insights into a better understanding, design, and construction of C1‐based cell factories for efficient C1 assimilation and high‐value product synthesis.

## 2 CARBON ASSIMILATION IN THE METABOLISM OF AEROBIC NATURAL C1‐UTILIZING BACTERIA

Natural C1‐utilizing bacteria can be either anaerobic (e.g., acetogens) or aerobic (e.g., methylotrophs). Anaerobic natural C1‐utilizing bacteria assimilate methanol or formate through an efficient linear Wood–Ljungdahl (WL) pathway and have notably higher product yield and energy efficiency than aerobic natural C1‐utilizing bacteria [1, 29]. However, the oxygen‐sensitive WL pathway relies on the carbon monoxide dehydrogenase/acetyl‐CoA synthase (CODH/ACS) to synthesize acetyl‐CoA from methyl group and carbon monoxide and is mostly found in some slow‐growing acetogens (<0.05 h^−1^) to generate a limited product spectrum such as short‐chain organic acids and alcohols (acetate, butyrate, and ethanol) [1, 30, 31]. For aerobic natural C1‐utilizing bacteria, methane is first converted to formaldehyde in two steps, the first catalyzed by the methane monooxygenase (MMO) and the second catalyzed by the methanol dehydrogenase (Mdh) [32]. The assimilation of formaldehyde may proceed through the ribulose monophosphate (RuMP) cycle [33], the serine cycle [34], or the newly discovered reductive glycine pathway [35]. Aerobic natural C1‐utilizing bacteria has relatively low energy efficiency as part of the electrons from carbon source enter the electron respiratory chain to reduce oxygen and generate water [1]. Although the production cost using aerobic natural C1‐utilizing bacteria is generally higher than anaerobic C1‐utilizing bacteria because of the requirement for continuous aeration and agitation, aerobic natural C1‐utilizing bacteria have advantages with higher growth rate (>0.1 h^−1^) and especially much broader product spectrum from single‐cell protein to diverse platform chemicals, fine chemicals, and biofuels due to the decoupling of product synthesis and energy conservation lifting thermodynamic constrains [1, 11].

### 2.1 The RuMP cycle for the formaldehyde assimilation

A quantitative understanding of the central carbon assimilation in the aerobic C1‐utilizing bacteria is essential for building efficient C1‐based cell factories that can synthesize diverse value‐added products. The RuMP cycle, which is advantageous over the serine cycle regarding energy and reducing power efficiency, can be associated with two different downstream pathways, the Entner–Doudoroff (ED) pathway and the Embden–Meyerhof–Parnas (EMP) pathway [4, 28]. In a representative methanotroph, *Methylococcus capsulatus* Bath, the activities of key enzymes in the ED pathway including 6‐phosphogluconate dehydrase and phospho‐2‐keto‐3‐deoxygluconate aldolase are 2.3‐fold higher than the key enzymes in the EMP pathway including phosphofructokinase (Pfk) and fructose diphosphate aldolase [36]. It is thus speculated that the assimilation of formaldehyde to generate pyruvate through the RuMP cycle mainly occurs via the ED pathway, while the EMP pathway only plays a secondary role. However, the RuMP cycle coupled with the ED pathway has low efficiency in ATP synthesis, and its predicted maximum carbon conversion rate (39%–47% based on quantitative calculation) is lower than the actual measured value (64%–66.5%) [24]. To solve this contradiction, Kalyuzhnaya et al. measured the temporal change of the ^13^C abundance of the different carbon atoms in pyruvate by transient ^13^CH_4_ tracing with LC‐MS and GC‐MS in *Methylotuvimicrobium alcaliphilum* 20Z, a haloalkalitolerant methanotroph and a promising C1 biocatalyst (Table 1) [24]. They found that the ^13^C incorporation rate into the third carbon of pyruvate is at least six‐fold higher than the incorporation rate into the first carbon, which confirmed that the major fraction of intracellular pyruvate is produced through the EMP pathway during growth on methane. According to further quantitative analyses of transcriptome and metabolome, the pyrophosphate (PPi)‐dependent Pfk is found to be responsible for the phosphorylation reaction in the first step of the EMP pathway (i.e., phosphorylation of fructose 6‐phosphate, F6P), and the transcriptome abundance of the EMP pathway‐related genes is 2–10 times higher than that of the ED pathway‐related genes. Stoichiometric analysis showed that the RuMP cycle is coupled with the PPi‐dependent EMP pathway to convert nine formaldehyde molecules into three molecules of 3‐phosphoglycerate (3PG), while generating two molecules of ATP and three molecules of NADH simultaneously. This ATP‐producing assimilatory route allows a microaerobic fermentation mode to produce a variety of excreted chemicals such as acetic acid, succinic acid, and lactic acid using methane as a feedstock. According to this discovery, Nguyen et al. introduced a heterologous 2,3‐butanediol (2,3‐BDO) synthetic pathway using pyruvate as the precursor into *M*. *alcaliphilum* 20Z [37]. They used a genome‐scale FBA model to predict the coupling relationship between cell growth and 2,3‐BDO biosynthesis, which suggested gene knockouts of *mdh*, *ack*, *ldh* responsible for pyruvate conversion to other intermediates via branched pathways. 2,3‐BDO was then synthesized with *M*. *alcaliphilum* 20Z fermentation mode under low oxygen (5%) supply, at a titer of 86.2 mg L^−1^ and a yield of 0.0318 g g^−1^ CH_4_. Using the genome‐scale FBA, Torre et al. found that *Methylotuvimicrobium buryatense* 5GB1 which has high growth rate on methane and methanol, also uses the RuMP cycle coupled with the EMP pathway to assimilate formaldehyde to synthesize pyruvate and its decarboxylated product acetyl‐CoA [38]. The yield of fatty acid methyl ester was thus enhanced to 111 ± 2 mg g^−1^ dry cell weight (DCW) in *M*. *buryatense* 5GB1 by blocking acetate synthesis from acetyl‐CoA and strengthening the expression of the downstream acetyl‐CoA carboxylase. According to the quantitative analysis of the central metabolism, Fei et al. further obtained a lipid productivity of 45.4 mg L^−1^ h^−1^ in *M*. *buryatense* 5GB1 in a continuous stirred tank bioreactor (CSTR), after optimizing methane and oxygen input rates and blocking glycogen synthesis from F6P [52].

**TABLE 1:** New insights of C1 metabolism for methane, methanol, and formate in representative C1‐utilizing bacteria have been studied by using various quantitative methods.

Quantitative methods	Representative C1‐utilzing bacteria	New insights of C1 metabolism	References
Transient ^13^C tracing, genome‐scale FBA, transcriptome, and metabolome	*M. alcaliphilum* 20Z; *M. buryatense* 5GB1	The RuMP cycle coupled with the EMP pathway is the major route to assimilate formaldehyde during growth on methane or methanol	[24, 37, 38]
^13^C isotopically nonstationary MFA, ^13^C isotopically steady state MFA based on two substrates (^13^C‐methane/methanol, ^12^C‐CO_2_, or vice versa), transcriptome, and metabolome	*M. buryatense* 5GB1	NADH mainly comes from the complete oxidation of methane; methanol metabolism has a higher carbon conversion efficiency than methane metabolism in generating methanotrophic biomass under limited concentration	[27, 39]
^13^C isotopically nonstationary MFA, and genome‐scale FBA	*Bacillus methanolicus* MGA3; *M. alcaliphilum* 20Z	The non‐oxidative pentose phosphate pathway is mainly used for ribulose 5‐phosphate generation	[40, 41]
Genome‐scale FBA, transient ^13^C tracing, ^13^C isotopically steady state MFA based on two substrates (^13^C‐methanol, ^12^C‐CO_2_, or vice versa), metabolome	*Methylorubrum* extorquens AM1	Quantifying flux partition at the formate branch point to the assimilative serine cycle and formate oxidation; glyoxylate regeneration is achieved through the ethylmalonyl‐CoA pathway; and constructing the synergistic methanol assimilation pathway	[22, 26, 42, 43]
^13^C isotopically steady state MFA based on two substrates (^13^C‐methanol, ^12^C‐CO_2_, or vice versa), ^13^C isotopically nonstationary MFA, and metabolome	*M. buryatense* 5GB1; *M. alcaliphilum* 20Z; *B*. *methanolicus* MGA3	The integrity of the TCA cycle determines whether it contributes to ATP, reducing power and generation of biomass precursors or only the precursor generation	[39, 40, 44, 45]
Genome‐scale FBA, ensemble modeling for robustness analysis, ^13^C labeling pattern analysis, transcriptome, and MMC	*E. coli‐*based synthetic methanol utilizer	Synthetic C1‐utilizing *E*. *coli* grown on methanol as the sole carbon and energy source by introduction of the RuMP assimilation cycle	[23, 46, 47, 48, 49]
Thermodynamic analysis and ^13^C labeling pattern analysis	*E*. *coli‐*based synthetic formate utilizer	Synthetic C1‐utilizing *E*. *coli* grown on formate and carbon dioxide as the carbon and energy source by introduction of the reductive glycine pathway	[50, 51]

Abbreviations: C1, one‐carbon; EMP, Embden‐Meyerhof‐Parnas; FBA, flux balance analysis; MFA, metabolic flux analysis; MMC, microbial microdroplet culture; RuMP, ribulose monophosphate.

**FIGURE 2:**
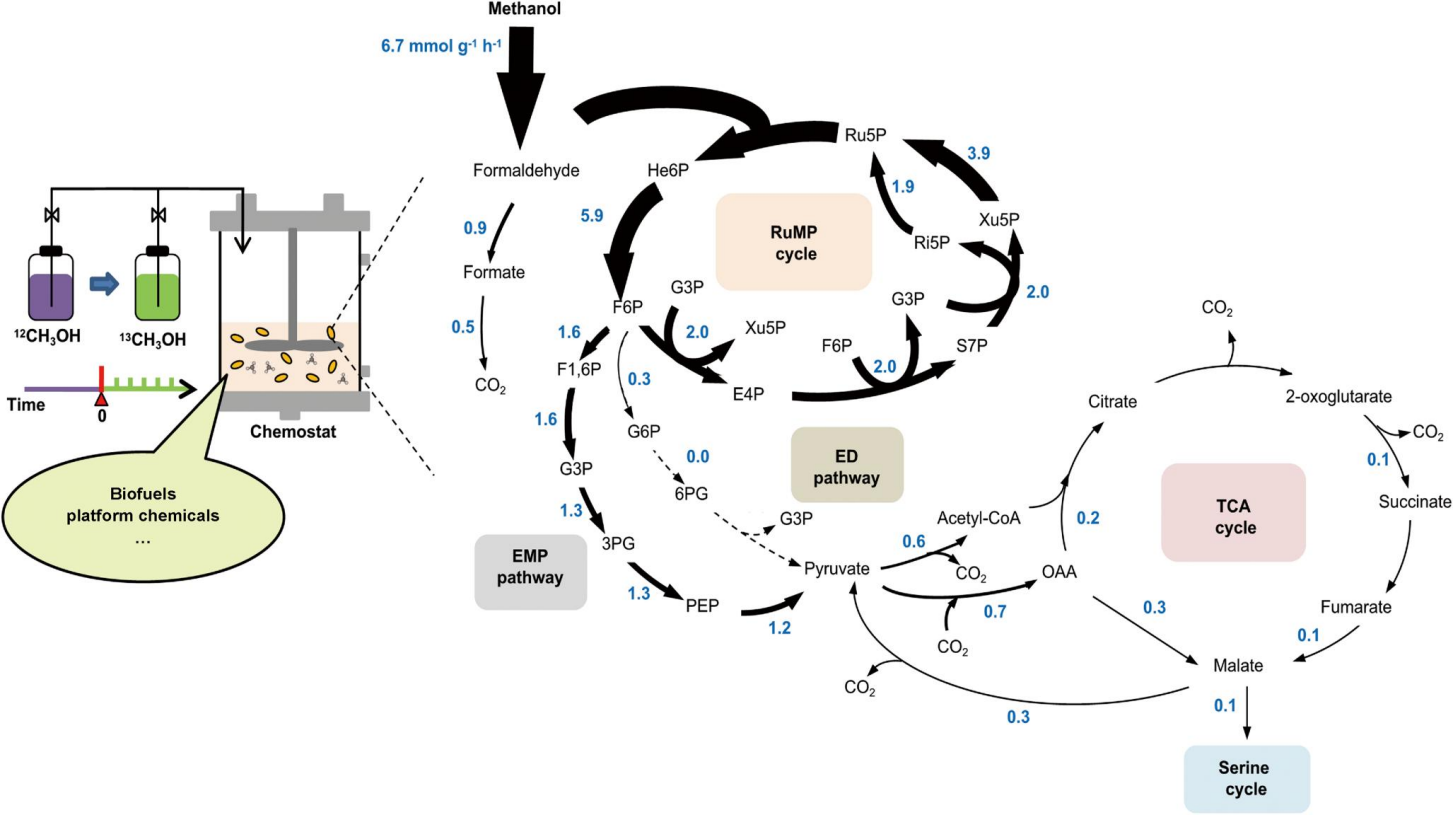
Revealing the central metabolic flux distribution of *M. buryatense* 5GB1C growing on methanol by ^13^C isotopically nonstationary metabolic flux analysis.^13^C isotopic experiment was conducted under a chemostat condition. At time point zero, the substrate was switched to ^13^C‐methanol (green) from ^12^C‐methanol (purple). The fluxes (blue number) were calculated under the dilution rate of 0.1 h^−1^. 3PG, 3‐phosphoglycerate; 6P, 6‐biphosphate; 6PG, 6‐phosphogluconate; E4P, erythrose‐4‐phosphate; ED, Entner‐Doudoroff; EMP, Embden‐Meyerhof‐Parnas; F1, fructose‐1; F6P, fructose‐6‐phosphate; G3P, glyceraldehyde‐3‐phospohate; G6P, glucose‐6‐phosphate; He6P, hexulose‐6‐phosphate; OAA, oxaloacetate; PEP, phosphoenolpyruvate; Ri5P, ribose‐5‐phosphate; Ru5P, ribulose‐5‐phosphate; RuMP, ribulose monophosphate; S7P, sedoheptulose‐7‐phosphate; TCA, tricarboxylic acid; Xu5P, xylulose‐5‐phosphate.

The oxidation of methane to methanol consumes the reducing power NADH, while the oxidation of methanol to formaldehyde generates either NADH or ATP when pyrroloquinoline quinone (PQQ) is used as a cofactor to transfer electrons to the respiratory chain. Therefore, natural C1‐utilizing bacteria show different energy and reducing power efficiencies when grown on methane or methanol. Whether they employ similar metabolic networks during growth on methane or methanol remains an open question. Traditionally, absolute metabolic fluxes can be calculated via a ^13^C isotopic steady state analysis method when multi‐carbon substrates such as glucose and glycerol are used as the feedstocks [53]. However, when C1 feedstocks are used, all intermediate metabolites derived from the ^13^C feedstocks are fully labeled, presenting a unique challenge to study C1 metabolic fluxes. He et al. recently developed a ^13^C isotopically nonstationary metabolic flux analysis (^13^C INST‐MFA) to quantify the central metabolic flux changes when methane and methanol are used as the carbon source (Figure 2, Table 1) [27]. They found that when methanol is used as a carbon source, the RuMP cycle coupled with the EMP pathway is the main pathway to assimilate formaldehyde, and less than 8% of the formaldehyde enters the tetrahydromethanopterin pathway, where they are oxidized to formate and eventually to carbon dioxide. Only 20% of the methanol is finally converted into carbon dioxide. In contrast, when methane is used as the carbon source, about 50% of the methane is eventually converted into carbon dioxide. For the first time, this work quantitatively demonstrates via ^13^C INST‐MFA that methanol has higher carbon conversion efficiency than methane in generating methanotrophic biomass under limited concentration conditions. In addition, Fu et al. studied the differences in transcriptome (RNA‐sequence) in *M. buryatense* 5GB1 when methane and methanol are used as carbon sources [39]. They found that the changes in gene expression of the central metabolism are less than 2 times, and thus speculated that the changes in metabolic flux distributions under methanol/methane growth conditions are mainly due to post‐transcriptional regulation.

As the RuMP cycle is an autocatalytic process, it needs sufficient ribulose 5‐phosphate (Ru5P) to react with formaldehyde to form F6P [54]. Ru5P can be regenerated through either the non‐oxidative pentose phosphate pathway (PPP) or the oxidative PPP [55]. Delépine et al. used ^13^C INST‐MFA to quantify fluxes when methanol is the carbon source for Gram‐positive methylotrophic bacterium *B. methanolicus* MGA3, which shows that 61% of F6P enters the non‐oxidative PPP to regenerate Ru5P, and only 9% of F6P enters the oxidative PPP for Ru5P (Table 1) [40]. Since the oxidative PPP produces carbon dioxide via the 6‐phosphogluconate dehydrogenase pathway, blocking this pathway theoretically facilitates more biomass generation from formaldehyde assimilation and thus more desired products generation. On the other hand, the oxidative PPP provides two molecules of reducing power NADPH required for cellular anabolism, making it essential when heterologous biosynthetic pathways with high NADPH demands are introduced in cells. By using genome‐scale FBA, Nguyen et al. found that, when *M*. *alcaliphilum* 20Z uses methane as the carbon source, about 75% of F6P enters the non‐oxidative PPP, which is higher than the corresponding ratio of *E*. *coli* and *Saccharomyces cerevisiae* using glucose as the carbon source [41]. By exploiting the non‐oxidative PPP of *M*. *alcaliphilum* 20Z, they obtained the mycosporine‐like amino acid shinorine at an initial titer of 0.52 mg L^−1^ on methane, whose key precursor is sedoheptulose 7‐phosphate (S7P). This titer is comparable to the shinorine production from *S*. *cerevisiae* using xylose as a carbon source (0.46 mg L^−1^), demonstrating the potential of using the non‐oxidative PPP of natural C1‐utilizing bacteria to synthesize high‐value products.

### 2.2 The serine cycle coupled to the EMC pathway for the formate assimilation

Formate, an oxidative product of formaldehyde can either be oxidized to CO_2_ by the formate dehydrogenase (Fdh), or be converted into formyl‐H_4_F by the formyltetrahydrofolate ligase (Fthfl) and subsequently be assimilated through the serine cycle into the biomass. Compared to the RuMP cycle, the serine cycle has a lower energy efficiency, requiring three molecules of NAD(P)H and three molecules of ATP to fix one molecule of formate and carbon dioxide for generating one molecule of acetyl‐CoA. Flux distributions show 10%–25% formate oxidized from formaldehyde enters the serine cycle in an extensively studied facultative methanol utilizer, *Methylobacterium* (also known as *Methylorubrum*) *extorquens* AM1 [22, 26, 56]. A biosensor‐assisted transcriptional regulator engineering for *M*. *extorquens* AM1 can further quantitatively increase the flux into acetyl‐CoA involved in the serine cycle by 7% [57]. Nevertheless, since *M*. *extorquens* AM1 and many other natural C1‐utilizing bacteria lack isocitrate lyase in the glyoxylate cycle, biomass generation through the serine cycle is obscured by the regeneration of glyoxylate, which is a key intermediate in the serine cycle [42]. Peyraud et al. designed transient ^13^C tracing analysis to prove that glyoxylate regeneration in *M*. *extorquens* AM1 is achieved through an ethylmalonyl‐CoA (EMC) pathway that continuously converts acetyl‐CoA molecules into a series of CoA derivatives (Table 1) [42]. After ^13^C‐acetate is added to *M*. *extorquens* AM1, eight CoA derivatives are labeled rapidly in succession, with acetyl‐CoA being the first to be labeled and succinate‐CoA the last. In the EMC pathway, two molecules of glyoxylate are regenerated from one molecule of acetyl‐CoA and two molecules of carbon dioxides, which in fact is a more efficient process than the classical glyoxylate cycle regarding carbon fixation. Subsequent transient ^13^C‐acetate tracing analysis also proved that *M*. *extorquens* AM1 mainly used the EMC pathway coupled with the TCA (tricarboxylic acid) cycle to assimilate acetate to generate biomass [58]. Since the CoA derivatives of the EMC pathway are important precursors for the formation of many carboxylic acids and alcohols (e.g., mevalonate, 3‐hydroxypropionic acid, and butanol) [59, 60, 61], Schada von Borzyskowski et al. proposed to construct the glyoxylate cycle in *M*. *extorquens* AM1 and block the original EMC pathway, in order to achieve cell growth dependence on the glyoxylate cycle and convert methanol to crotonic acid [62].

### 2.3 The TCA cycle for ATP, reducing power or biomass precursors generation

**FIGURE 3:**
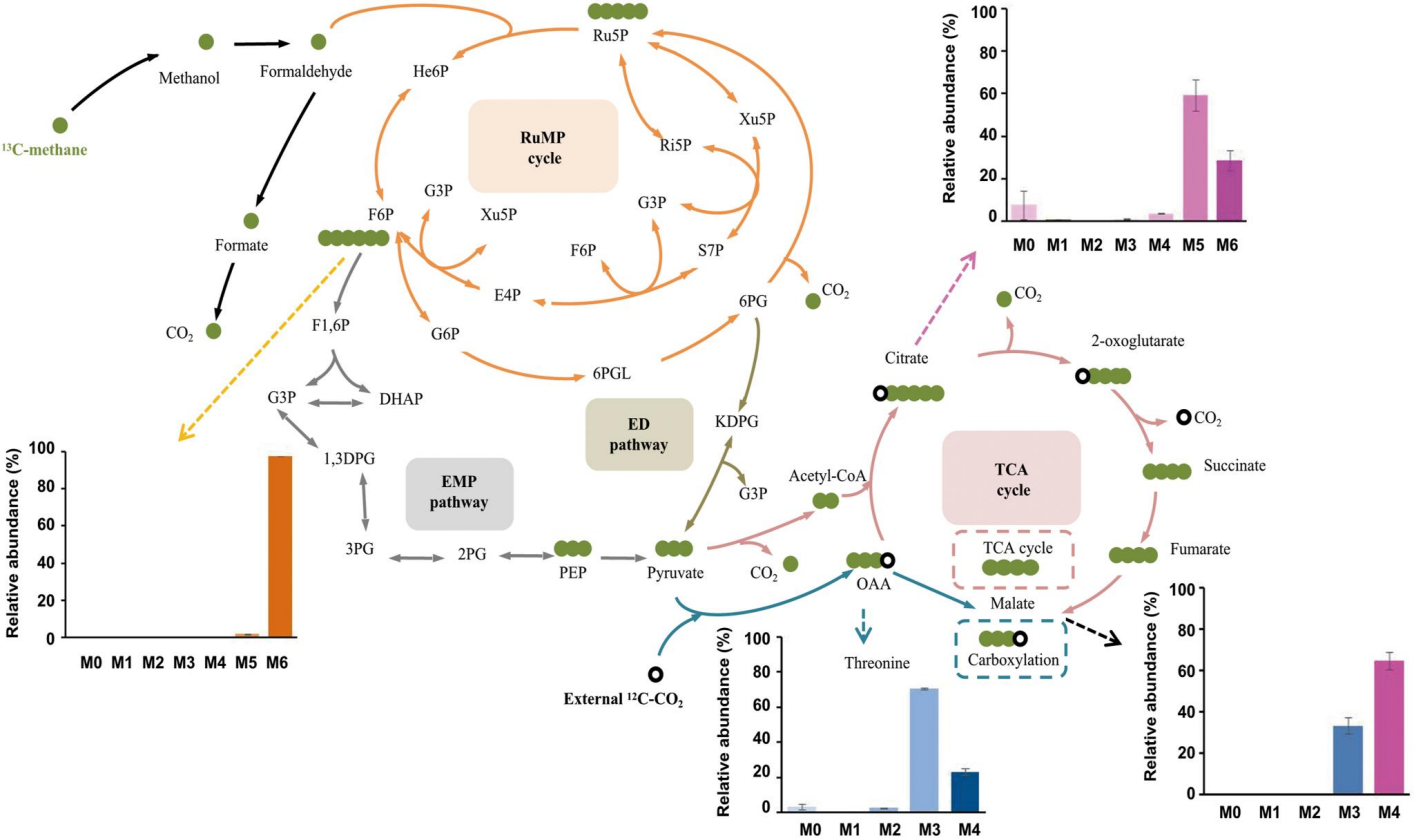
Mapping carbon atomic transition of the central methanotrophy in *M. buryatense* 5GB1 growing on ^13^C‐methane under metabolic and isotopic steady state with ^12^C‐CO_2_ carboxylation. 1,3DPG, 2,3‐bisphosphoglyceric acid; 2PG, 2‐phosphoglycerate; 3PG, 3‐phosphoglycerate; 6PG, 6‐phosphogluconate; 6PGL, 6‐phosphogluconolaetone; DHAP, dihydroxyacetone phosphate; E4P, erythrose‐4‐phosphate; ED, Entner–Doudoroff; EMP, Embden–Meyerhof–Parnas; F1,6P, fructose‐1,6‐biphosphate; F6P, fructose‐6‐phosphate; G3P, glyceraldehyde‐3‐phospohate; G6P, glucose‐6‐phosphate; He6P, hexulose‐6‐phosphate; KDPG, 2‐keto‐3‐deoxy‐6‐phosphogluconate; OAA, oxaloacetate; PEP, phosphoenolpyruvate; Ri5P, ribose‐5‐phosphate; Ru5P, ribulose‐5‐phosphate; RuMP, ribulose monophosphate; S7P, sedoheptulose‐7‐phosphate; TCA, tricarboxylic acid; Xu5P, xylulose‐5‐phosphate.

The TCA cycle is an important pathway for many heterotrophic microorganisms to generate ATP, reducing power, and precursor metabolites. Early studies of *M*. *buryatense* 5GB1 did not identify 2‐oxoglutarate dehydrogenase, a key enzyme in the TCA cycle, and could not verify the integrity and function of the TCA cycle. Recently Fu et al. designed a ^13^C steady state MFA to examine the labeling patterns of malate, fumarate, and citrate in the TCA cycle of *M*. *buryatense* 5GB1 cultured in ^13^C‐methane and to compare the differences of these downstream metabolites when phosphopyruvate and pyruvate participate in the carboxylation reaction (Figure 3, Table 1) [44]. The design principle of this ^13^C steady state MFA is similar to the co‐substrate experiment, in which a C1 substrate is applied along with a second substrate (herein is CO_2_), where one is ^13^C labeled and the other is unlabeled. This ^13^C‐MFA experiment simplifies the experimental setup and data analysis as it can be conducted at both metabolic and isotopic steady state. Based on the labeling patterns, Fu et al. found that malate showed higher fraction of fully labeled isotopologue than threonine, which suggested that 45% of malate is synthesized from fumarate through the TCA cycle, thus proving a functional, complete oxidative TCA cycle providing not only precursor metabolites but also ATP and reducing power for biomass synthesis. However, when methanol is used as the carbon source for *M*. *buryatense* 5GB1 and *M*. *alcaliphilum* 20Z, triply‐labeled malate is of higher abundance than fully‐labeled malate, indicating that malate mainly comes from pyruvate or oxaloacetate instead of fumarate [39, 45]. That is, the oxidative TCA cycle is incomplete when methanol is used as the carbon source, and thus it only provides the precursor metabolites. These results again show that methane generates more reducing power and ATP level than methanol. In addition, according to the ^13^C INST‐MFA, the TCA cycle in *B. methanolicus* MGA3 operates in both oxidative and reductive fashions when grown on methanol [40]. Such bifunctional TCA cycle may inspire further optimization of methylotrophic cell factories that produce the derivatives of the TCA cycle intermediate metabolites (e.g., l‐glutamate [63]).

Methane was used as ^13^C‐substrate. ^13^C‐labeled carbon atoms were displayed using solid green circles and the carbon atoms with external ^12^C‐CO_2_ using hollow black circles. There are two possibilities to synthesize malate: either by carboxylation pathway (blue) or through the TCA cycle according to the network topology and atomic transition. C3 metabolites and intermediates involved in the RuMP cycle are fully labeled.

## 3 REWIRING THE METABOLIC MODULES OF NATURAL C1‐UTILIZING CELL FACTORIES TO IMPROVE PERFORMANCE

The quantitative analysis of the assimilation process of natural C1‐utilizing bacteria guides the rational remodeling of the assimilation module. The conversion yield of formaldehyde into acetyl‐CoA is only 67% through the RuMP cycle when coupled with either the EMP or the ED pathway, as pyruvate dehydrogenase leads to one carbon lost as carbon dioxide [64]. This major problem is circumvented in the serine cycle, in which acetyl‐CoA is synthesized via a breakdown of malyl‐CoA without carbon loss [34]. To improve the carbon conversion efficiency, Yuan et al. rewired the assimilation pathway by integrating both the RuMP cycle and the serine cycle into *M*. *extorquens* AM1, which allows the synergistic assimilation of formaldehyde and formate [43]. Different metabolic flux ratios of the RuMP cycle to the serine cycle in *M*. *extorquens* AM1 were simulated by a genome‐scale FBA model, which predicted a theoretical maximum growth rate up to 0.281 h^−1^. The synergistic assimilation chassis constructed based on the FBA model prediction gave a 9.0% increase in specific growth rate and 2.1 times increase in the acetyl‐CoA pool. The metabolome and transcriptome analysis indicated that the acetyl‐CoA mainly came from the cleavage of malyl‐CoA in the serine cycle, which avoids carbon loss from decarboxylation. When the heterologous pathway of 3‐hydroxypropionic acid (3‐HP) synthesis was introduced into the *M*. *extorquens* AM1 mutant harboring both the RuMP cycle and the serine cycle, the resulting cell factory produced 3.1 times higher 3‐HP than the *M*. *extorquens* AM1 mutant expressing only the 3‐HP pathway. After 100 h of cultivation by fed‐batch fermentation, the maximum titer of 3‐HP was up to 0.857 g L^−1^ with the yield of 0.031 g g^−1^ methanol.

The phosphoketolase (PKT) pathway includes a phosphoketolase (Pkts) catalyzing the irreversible phosphorylytic cleavage of xylulose 5‐phosphate (Xu5P) and/or F6P to acetyl‐phosphate and glyceraldehyde‐3‐phosphate or erythrose‐4‐phosphate, respectively [65]. Then the acetyl‐phosphate is readily converted to acetyl‐CoA by either acetate kinase (Ack) combined with acetyl‐CoA synthetase (Acs) or phosphotransacetylase (Pta) alone, which does not have carbon loss. In many natural C1‐utilizing bacteria such as *M*. *alcaliphilum* 20Z, *M*. *capsulatus* Bath, and *M*. *extorquens* AM1, the genes of *pkt*, *pta*, *ack*, or *acs* are found to be present on the chromosome, supporting the potential physiological role in the production of acetyl‐CoA and/or acetate [66]. Based on the advanced carbon yield of the PKT pathway, Bogorad et al. designed a methanol condensation cycle (MCC) [67], by combining the PKT pathway with the RuMP cycle to convert methanol to alcohols using in vitro enzymatic reactions. ^13^C tracing from ^13^C formaldehyde to ^13^C ethanol demonstrated that the MCC is a complete catalytic cycle. After 5‐h reactions, the MCC led to a final titer of 610 mg L^−1^ ethanol from 1072 mg L^−1^ methanol. The molar yield of carbon was 80%, exceeding the theoretical yield from the RuMP cycle coupled to the EMP pathway. Furthermore, Henard et al. overexpressed the PKT pathway in *M*. *buryatense* 5GB1 to synthesize acetyl‐CoA in the RuMP cycle‐based methanotrophs [68]. Because carbon loss is avoided in the PKT pathway, the remodeled methanotrophic chassis have 2 times larger acetyl‐CoA pool, 2.6 times greater conversion yield from methane to biomass, and 2 times higher lipid titer.

A number of natural C1‐utilizing bacteria possess genes and enzymes of the Calvin–Benson–Bassham (CBB) cycle; however, the contribution of the CBB cycle to methylotrophy remains poorly understood [32]. Recently, Henard et al. used isotopic tracing with ^12^C‐methane and ^13^C‐CO_2_ as mixed carbon sources to prove that *M*. *capsulatus* Bath uses the methylotrophic pathway to assimilate methane while using the CBB cycle to fix carbon dioxide [69]. Accordingly, to increase the efficiency of carbon fixation, Schada von Borzyskowski et al. designed a chassis to decouple the methanol assimilation pathway and dissimilation pathway in *M*. *extorquens* AM1 [70]. In the constructed autotrophic *M*. *extorquens* AM1, the carbon needed for the biomass synthesis comes solely from carbon dioxide, whereas the energy and reducing power comes from the oxidation of methanol. Transient ^13^C‐carbonate tracing proves that the constructed autotrophic methylotroph quickly fixes carbon dioxide through the CBB cycle, and the future adaptive evolution is expected to further enhance the efficiency of CBB cycle in fixing carbon dioxide. This work provides a novel insight into engineering the methylotrophic or methanotrophic bacteria to synthesize high‐value products using both organic and inorganic C1 substrates.

The vulnerability of natural C1‐utilizing bacteria to high concentration of methanol impedes its further development as a chemical‐producing platform. Improvement of methanol tolerance through rational design requires substantial knowledge on C1 assimilation modules, as well as molecular mechanisms of cell response to methanol toxicity, which could be complicated as revealed in *M*. *extorquens* strains and other bacteria [46, 71]. Conventional adaptive laboratory evolution (ALE) is time consuming with inefficient mutation rate. Recently, Cui et al. utilized atmospheric and room temperature plasma (ARTP) mutagenesis, in combination with ALE to accelerate the mutation rate [72]. Within a short time of cell population transfers, an evolved *M*. *extorquens* AM1 (CLY‐2533) was generated to display high methanol tolerance, whose cell density was 7.1‐fold higher than that of the parent strain grown on 5% (v/v) methanol. Accordingly, the mevalonate volumetric productivity of the evolved strain carrying a mevalonate synthetic pathway was 65% higher than that of the parent strain in methanol fed‐batch fermentation. Combined with whole genome sequencing and genetic basis testing, the phenotype–genotype correlations of improving methanol tolerance was further identified in evolved *M*. *extorquens* AM1, which can be used as a blueprint for rewiring the metabolic modules of natural C1‐utilizing bacteria and other industrial chassis strains in the future.

## 4 CREATING SYNTHETIC C1‐UTILIZING CELL FACTORIES

In recent years, creating synthetic C1‐utilizing bacteria by introducing the highly efficient RuMP cycle to broadly studied and industrially promising non‐C1‐utilizing bacterial strains has been a new research focus, which requires overexpression of only three heterologous genes, that is, methanol dehydrogenase (*mdh*), 3‐hexose phosphate synthase (*hps*), and 6‐phosphate‐3‐hexose isomerase (*phi*) [73]. Meyer et al. adopted a methanol auxotrophy strategy by expressing the above three genes in *E*. *coli* and knocking out genes related to multi‐carbon source utilization, which forces the reconstructed cell chassis to depend on methanol assimilation for growth and thus creates a selection pressure for the following adaptive direct evolution to eventually achieve a methanol‐essential *E*. *coli* strain [74]. Wang et al. and Jian et al. then developed a novel irrational strategy based on an automatic high‐throughput selection of microbial microdroplet culture (MMC) platform to evolve the methanol‐essential *E*. *coli* strain [47, 48]. One of the evolved strains (MeSV2.2‐3) was achieved with a 3‐fold faster growth rate, a 43% shorter lag phase, and 40% less co‐substrate of gluconate usage compared with the parent strain, showing that a promising C1‐utilizing chassis strain being more dependent on methanol can be achieved using the MMC system. Furthermore, Keller et al. used genome‐scale FBA model to populate a list of 1200 methanol growth‐dependent strains that have a complete RuMP cycle (Table 1) [23]. These 1200 strains were then ranked based on two metrics to determine their potential as a starting chassis for adaptive evolution, that is, the content of methanol‐derived biomass in the predicted strain and the dependence of the RuMP cycle on methanol assimilation. Of the two strains selected for experimental verification, one lacks fructose‐1,6‐bisphosphatase (Fbp) and the other lacks triosephosphate isomerase (Tpi), with both having a complete RuMP cycle and capable of utilizing methanol efficiently. Isotopic labeling patterns of these two strains with 13C‐methanol and 12C‐pyruvate as the mixed carbon source showed that 31% and 99% of the 13C‐methanol entered the RuMP cycle, respectively, and the observed high dependence of cell growth on methanol provided an ideal starting point for achieving a synthetic methanol‐utilizing bacterium in following the laboratory evolution. Based on these results, Keller et al. evolved methanol‐dependent *E*. *coli* in a chemostat with methanol and pyruvate as the mixed carbon source and, by continuously decreasing the amount of pyruvate over 250 generations of evolution, obtained an evolved mutant (MEcoli_ref_1) that used methanol as the sole carbon source and energy source [49]. This mutant had a doubling time of 8.1 h, and the biomass accumulation was completely derived from methanol as confirmed by the protein amino acid labeling pattern analysis using ^13^C‐methanol and ^12^C‐CO_2_. In a parallel study, Chen et al. analyzed the kinetic trap of engineered synthetic C1‐utilizing *E*. *coli* with ensemble modeling for robustness analysis (EMRA) and found that the autocatalytic RuMP cycle can be improved by knocking down the enzymatic activity of Pfk and glyceraldehyde 3‐phosphate dehydrogenase (GAPDH) involved in the glycolytic pathway [46]. They also obtained an evolutionary mutant (SM1) grown solely on the methanol through adaptive evolution with a doubling time of about 8 h. Moreover, Woolston et al. designed an isotopic tracing experiment based on the kinetic isotope effect using deuterated methanol as the carbon source and showed that in the presence of a sufficient supply of Ru5P, the enzyme activity of MDH is the major limiting factor for the growth of synthetic C1‐utilizing bacteria [75]. In addition to building the RuMP cycle in non‐C1‐utilizing bacteria, Yu et al. engineered *E*. *coli* by introducing a modified serine cycle to increase the carbon conversion efficiency from formate to acetyl‐CoA [76]. The engineered strain could produce 36.7 mM ^13^C‐acetate from the mixed carbon source containing 30 mM xylose, 200 mM ^13^C‐methanol, and 20 mM ^13^C‐carbonate, thus demonstrating the ability of the modified serine cycle to assimilate methanol and fix carbon dioxide in non‐C1‐utilizing bacteria. In addition, Lu et al. and Schwander et al. calculated the Gibbs free energy of the carbon fixation pathway and considered carboxylase kinetics of related reactions to design the pyruvate carboxylase/oxaloacetate acetylhydrolase/pyruvate:ferredoxin oxidoreductase/acetate‐CoA ligase (POAP) cycle and crotonyl‐CoA/ethylmalonyl‐CoA/hydroxybutyryl‐CoA (CETCH) cycle for achieving carbon dioxide fixation in vitro, respectively [77, 78]. Theoretically, these two pathways can also function for methanol and formate as the reduced C1 substrate when their thorough oxidation generates reducing power and ATP to drive the reassimilation of carbon dioxide. Although currently it is challenging to build up these synthetic networks in vivo because of the interference with the complex context of the host microorganism, these in vitro synthetic pathways still provide the attractive references for constructing synthetic C1‐utilizing bacteria in the future.

**FIGURE 4:**
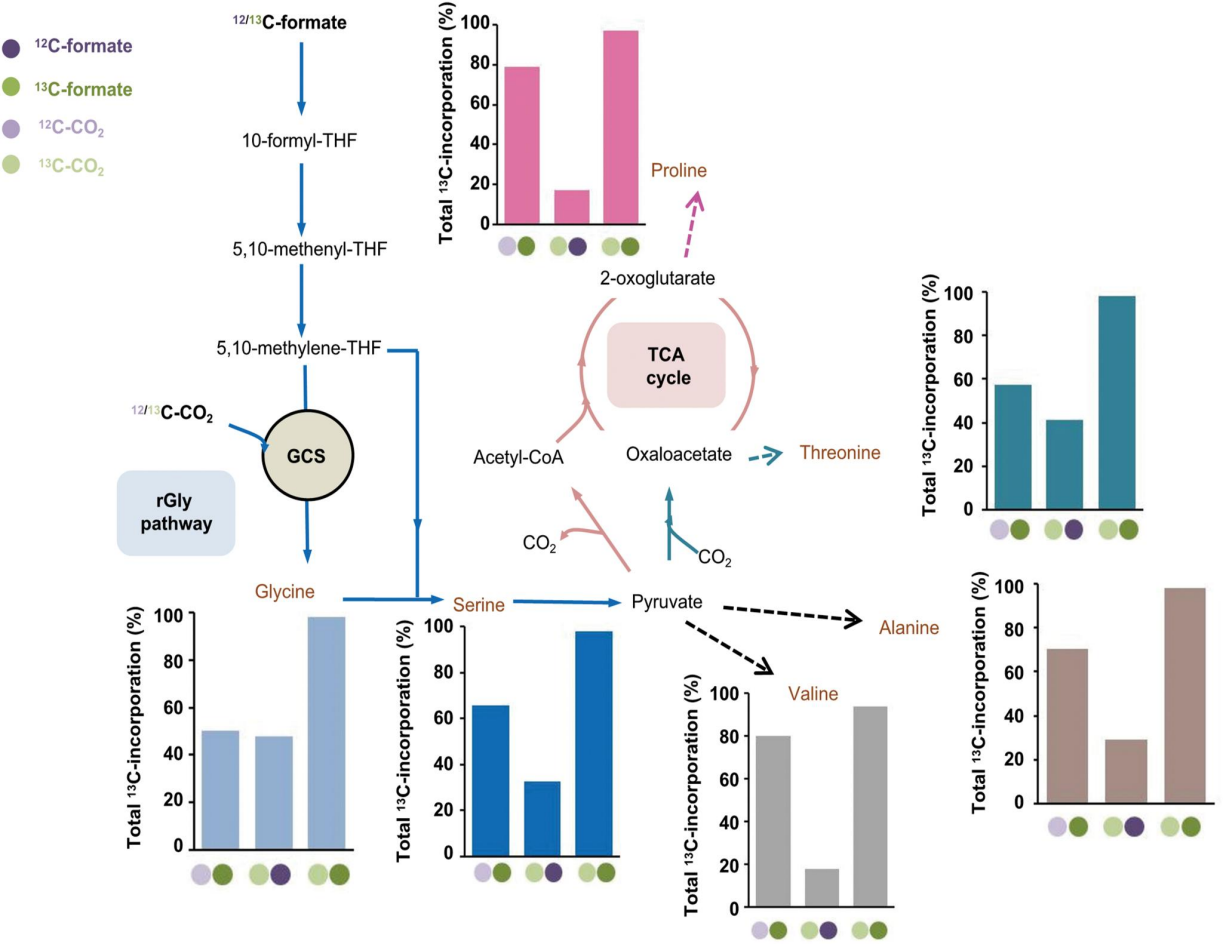
Quantitatively analyzing the ^13^C labeling patterns under different labeled tracers to confirm that the reductive glycine pathway fully support the growth of synthetic non‐C1‐utilizing bacteria of *E. coli* on the formate and carbon dioxide. Dark green, ^13^C‐formate; Dark purple, ^12^C‐formate; GCS, glycine cleavage system; light green, ^13^C‐CO_2_; light purple, ^12^C‐CO_2_; rGly, reductive glycine; TCA, tricarboxylic acid; THF, tetrahydrofolate.

In recent years, researchers have also focused on constructing the linear one‐carbon assimilation pathways in which no carbon has to be retained within an autocatalytic cycle. Bar‐even et al. designed a reductive glycine (rGly) pathway, which was later discovered to already exist in *Desulfovibrio desulfuricans* (Figure 4) [35], and predicted that this pathway can be the most efficient formate assimilation pathway under aerobic conditions by comparing the theoretical biomass yield, ATP consumption, and reaction thermodynamics among various formate assimilation pathways [50]. Compared to the WL pathway, the rGly pathway has a similarly high efficiency but is not oxygen sensitive. They then constructed serine auxotrophic *E*. *coli* and quantified the ^13^C labeling patterns of the main amino acids using mixed tracers, ^13^C‐formate and ^12^C‐CO_2_, ^12^C‐formate and ^13^C‐CO_2_, or ^13^C‐formate and ^13^C‐CO_2,_ to verify that the engineered *E*. *coli* could assimilate formate to synthesize serine via the rGly pathway (Figure 4, Table 1) [51]. Next, by strengthening the formate dehydrogenase via adaptive evolution, they ultimately obtained a synthetic formate and carbon dioxide‐utilizing *E*. *coli* that has the doubling time of 8 h and gives the biomass yield of 2.3 g DCW mol^−1^ formate. These achievements accompanied by metabolomic analysis of glycine‐related metabolic pathways [79] have encouraged the researchers to introduce the rGly pathway to other bacteria such as *Cupriavidus necator* [80], *Pseudomonas putida* [81], and *Clostridium pasteurianum* [82] to create synthetic strains that can assimilate formate. In addition, other novel linear pathways have been designed based on computational reaction thermodynamics, enzyme kinetics, and minimization of reaction steps, which theoretically outperform natural pathways (e.g., the RuMP cycle) in various aspects (e.g., ATP and reducing power production, carbon conversion, etc.) [83, 84]. These designs were validated by in vitro experiments. For example, Siegel et al. use RosettaDesign and Foldit to design a mutant benzaldehyde lyase, referred to as formolase, which has enhanced affinity toward formaldehyde [85]. The formolase catalyzes the one‐step conversion of three molecules of formaldehyde to one molecule of dihydroxyacetone, and the catalytic efficiency of *k*_*cat*_/*K*_*M*_ reaches 4.7 M^−1^ s^−1^. These new designs of linear C1 assimilation pathway provide another solution for further improving the cell growth performance and product synthesis in synthetic C1‐utilizing bacteria in the future.

## 5 SUMMARY AND OUTLOOK

The early studies of the metabolism of natural C1‐utilizing bacteria do not offer direct confirmation of metabolic precursors and give accurate quantitative descriptions of the metabolic flux distribution. Recent advances in novel techniques, such as isotopically nonstationary MFA and isotopic steady‐state analysis based on mixed carbon sources, have resolved many prior limitations and enabled a deeper understanding of the metabolic networks of natural C1‐utilizing bacteria. These new knowledge reveals promising metabolic pathways that can be targeted for production of non‐native metabolites via genetic manipulation of native pathways and introduction of heterogeneous metabolic routes [86]. These quantitative analyses also reveal metabolic bottlenecks, rate‐limiting steps, or carbon‐loss reactions, which can be overcome by the introduction of a new, energy‐efficient pathway either from other microorganisms, or through rational computational designs, or a novel adaptive evolution method. In addition, useful tools for genetic manipulation have been developed in native C1‐utilizing bacteria, including inducible promoters (e.g., isopropyl β‐D‐Thiogalactoside‐inducible promoter P_L/O4_ and levulinic acid‐inducible promoter P_hpdH_) [87, 88], the CRISPR interference (CRISPRi) technology [89, 90] and small‐molecule biosensors [57], which can all be employed to control gene expression. The combined hyper‐saturated CRISPR‐Cas9 [91] or transposon mutagenesis [71] with high‐throughput sequencing have also extended our understanding of methylotrophy under different growth conditions. Together, these quantitative techniques in metabolic network analysis, gene expression regulation, and functional regulator discovery will further help rationally engineer the synergistic C1 assimilation pathways with greater precision and efficiency in coupled cell growth and high‐value products synthesis. Notably, the study on biocatalyst of the methylotrophic yeast to assimilate methanol through the xylulose monophosphate cycle has achieved rapid progress in recent years [92, 93, 94]. Starting from the lethal phenotype of fatty acid‐producing yeast *Ogataea polymorpha*, Gao et al. identified the key mechanism for strengthening methanol tolerance through rational pathway engineering, adaptive evolution, and omics quantitative analysis, respectively, and they achieved a high‐level production of fatty acid of 15.9 g L^−1^ from methanol solely [95]. These two chassis of natural C1‐utilizing bacteria and yeast are complementary, and together they will help contribute to enhancing C1‐based biomanufacturing for renewable biofuel and chemical production.

Efforts have been made to introduce the RuMP cycle and the rGly pathway into *E*. *coli*, to create synthetic C1‐utilizing bacterial strains that use methanol and formate as the sole carbon and energy sources. Nevertheless, the growth rate is lower than that of aerobic natural C1‐utilizing bacteria and similar to that of anaerobic natural C1‐utilizing bacteria, and the biosynthesis of high‐value products is rarely reported [96]. It can be expected that synthetic C1‐utilizing bacteria can be further engineered to a more efficient cell factory producing a broad spectrum of products, as abundant metabolic engineering studies have been carried out on *E*. *coli*.

Due to the rapid increase of heat‐trapping greenhouse gases such as carbon dioxide and methane in the atmosphere, developing new technologies to enable greenhouse gas mitigation and value‐added chemical production simultaneously is imperative. Just recently, Cai et al. implemented the concept of combined carbon dioxide electrochemical catalysis and methanol bioconversion, and they reported in vitro starch biosynthesis using carbon dioxide as the carbon source and hydrogen as the energy source, which was enabled though assimilation pathway design, module assembly, and enzyme engineering [97]. As a result, they attained a significantly high carbon dioxide fixation rate at 22 nmol CO_2_ min^−1^ mg^−1^ total catalyst. This breakthrough research not only makes it possible to transform starch production from the traditional agricultural planting mode to an industrial workshop production mode, but also opens up new possibilities for the synthesis of complex molecules from carbon dioxide. In future, we expect that the automated, high‐throughput platform for growth‐coupled adaptive evolution such as microbial microdroplet cultivation and screening system will provide new ideas for the rational design of synthetic C1‐utilizing cell chassis. Combining with other strategies, such as computational pathway design, advanced metabolic flux analysis, and enzyme engineering will further lead to significant improvements for integrating novel C1‐assimilation pathways into synthetic cell factories for synthesis of diverse high value‐added products.

## AUTHOR CONTRIBUTIONS

**Yazhen Song**: Investigation; writing – original draft. **Chenxi Feng**: Investigation; writing – original draft. **Difei Zhou**: Investigation; writing – original draft. **Zengxin Ma**: Investigation; writing – original draft. **Lian He**: Writing – review and editing. **Cong Zhang**: Writing – review and editing. **Guihong Yu**: Writing – review and editing. **Yan Zhao**: Writing – review and editing. **Song Yang**: Conceptualization; writing – review and editing; funding acquisition; supervision. **Xinhui Xing:** Conceptualization; writing – review and editing; funding acquisition; supervision.

## CONFLICT OF INTEREST STATEMENT

Yazhen Song, Chenxi Feng, Difei Zhou, Zengxin Ma, Lian He, Cong Zhang, Guihong Yu, Yan Zhao, Song Yang, and Xinhui Xing declare that they have no conflict of interest.

## ETHICS STATEMENT

This article is a review article and does not contain any studies with human or animal subjects performed by any of the authors.
